# Similarities in the evolution of plants and cars

**DOI:** 10.1371/journal.pone.0198044

**Published:** 2018-06-29

**Authors:** Samantha Hartzell, Mark Bartlett, Jun Yin, Amilcare Porporato

**Affiliations:** 1 Department of Civil and Environmental Engineering, Princeton University, Princeton, NJ, United States of America; 2 Princeton Environmental Institute, Princeton University, Princeton, NJ, United States of America; 3 Department of Civil and Environmental Engineering, Duke University, Durham, NC, United States of America; Chongqing University, CHINA

## Abstract

While one system is animate and the other inanimate, both plants and cars are powered by a highly successful process which has evolved in a changing environment. Each process (the photosynthetic pathway and the car engine, respectively) originated from a basic scheme and evolved greater efficiency by adding components to the existing structure, which has remained largely unchanged. Here we present a comparative analysis of two variants on the original C3 photosynthetic pathway (C4 and CAM) and two variants on the internal combustion engine (the turbocharger and the hybrid electric vehicle). We compare the timeline of evolution, the interaction between system components, and the effects of environmental conditions on both systems. This analysis reveals striking similarities in the development of these processes, providing insight as to how complex systems—both natural and built—evolve and adapt to changing environmental conditions in a modular fashion.

## Introduction

Today, plants make up the majority of living biomass on earth [1] and the automobile is by far the most popular method of passenger transport worldwide [2]. These systems, while quite different in their functions, are powered by processes which have evolved over time in a remarkably similar fashion. Both originated from a basic, highly successful scheme and improved by adding components in a process of modular evolution. Designers of cars limited by oxygen availability developed the turbocharger [3], which functions similarly to the C4 “carbon pump” by concentrating a limiting reactant to improve efficiency [4]. As demand for fuel and water use efficiency increased, designers introduced the energy storage system of the Hybrid Electric Vehicle (HEV) to address inefficiencies caused by variable power demand [5], while plants evolved the Crassulacean Acid Metabolism (CAM) carbon storage system to reduce inefficiencies caused by diurnal variability in light and atmospheric humidity [4].

**Fig 1:**
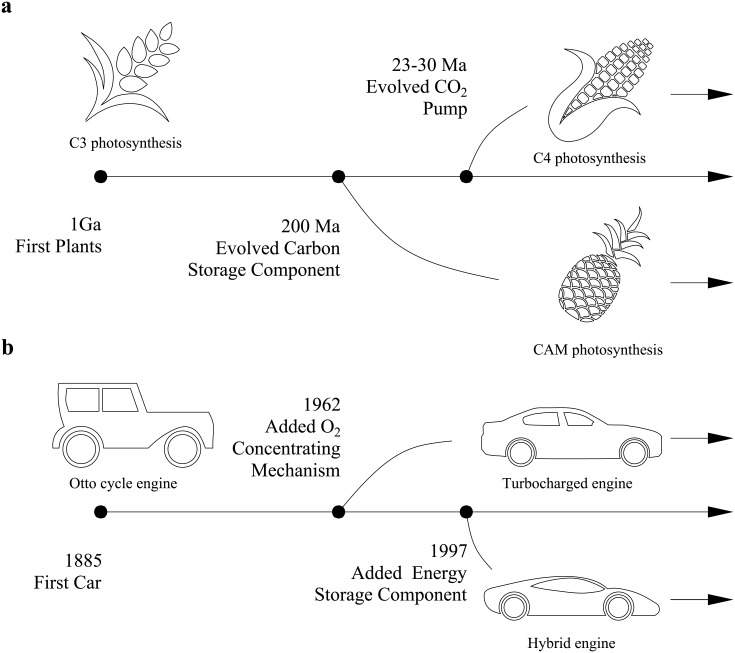
Comparative evolution of plants and cars. (a) In 1885, Karl Benz was among the automobile’s first producers, and in 1908, the Ford Motor Company pioneered the first mass produced automobile, the Model T [5]. The turbocharger gained popularity during World War II, when it was used in military aircraft, which had to cope with low-pressure, high-altitude air [3], and the first turbocharged passenger car, the Chevrolet Corvair Monza, debuted in 1962 [10]. Serious interest in hybrid technology arose in the 1960s when it was recognized as a means for harnessing variability in driving conditions to lower fuel use and emissions, and the Toyota Prius was introduced in 1997 as the first mass produced hybrid car [5]. (b) The first C3 plants developed around 1 Ga ago as aquatic lifeforms [6]. CAM photosynthesis evolved during the Paleozoic era and likely experienced a significant expansion in terrestrial plants in the Cenozoic era, which was accompanied by increasing seasonality of water availability [4]. C4 photosynthesis is thought to have first evolved in the mid-Tertiary period and experienced a large increase in the late Miocene, 4-7 Ma, which brought decreasing CO_2_ levels [4].

The basic photosynthetic pathway uses light energy to transform carbon dioxide into three-carbon sugars which are used to power plant processes and build tissue. This is accomplished through a complex series of processes involving the light reactions, which use light energy to break up water into oxygen and protons (fuel), and the Calvin cycle, which fixes carbon dioxide into sugar (energy). The C3 pathway, so-called because of the three-carbon sugar it produces, was the first photosynthetic pathway to evolve in modern terrestrial plants. According to the endosymbiotic theory, this pathway developed in eukaryotes around 1 Ga ago when photosynthetic cyanobacteria were first incorporated into algae as chloroplasts. This development was then carried over into terrestrial plants [6]. The basic C3 pathway can be compared to the modern Otto cycle internal combustion engine (ICE), which was patented by Nikolaus Otto in 1876. Like the revolution caused by the incorporation of photosynthetic bacteria into algae, this gasoline engine was quickly incorporated into the first automobiles (see Fig 1 for a brief evolutionary history of both systems). Since then, many aspects of the automobile design have changed, but the main agent of propulsion, the ICE, has remained remarkably consistent [7], as has the chloroplast in plants [8, 9].

**Fig 2:**
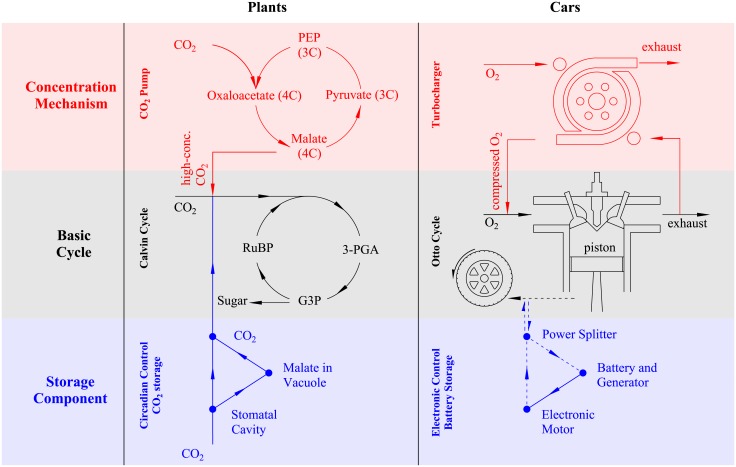
A comparison of plant photosynthesis and car engine functioning illustrates how the core processes interact with the additional components. The core processes in each system are the Calvin cycle and the ICE (middle row). A concentrating mechanism in C4 plants and turbocharged cars provides concentrated CO_2_ and oxygen, respectively, to the core cycle (upper row). A storage mechanism in CAM plants allows carbon dioxide to be stored as malic acid at night and then passed to the Calvin cycle during the day, while a storage mechanism in HEVs allows energy to be stored in the battery during braking and then passed to the motor to power the drivetrain in parallel with the engine (bottom row).

Both the Otto cycle and the C3 photosynthetic pathway have limited efficiency under typical operating conditions. The internal combustion engine works by combusting fuel with an oxidizer (air). The power produced is limited, among other factors, by the amount of air taken into the engine. This is characterized by the volumetric efficiency, i.e., the ratio of the actual to the theoretical maximum amount of air which could be taken in [7]. ICEs also experience a large decrease in fuel efficiency under variable traveling speed (particularly stop-and-go traffic), as the engine is constantly running and the braking process dissipates kinetic energy. In the C3 photosynthetic pathway, carbon dioxide diffuses into the leaf and reacts with ribulose-l,5-bisphosphate (RuBP) to produce sugars, which are ultimately used to form carbohydrates (see Fig 2). Efficiency is strongly impacted by photorespiration, a process by which RuBP reacts with oxygen, rather than carbon dioxide. Under the modern atmospheric composition, the high concentration of oxygen relative to carbon dioxide leads to significant photorespiration, reducing the overall efficiency of C3 plants by about one-third [11]. Plant efficiency is also limited by considerations of water availability. Water use efficiency, i.e., the ratio of carbon assimilated to water vapor lost, is a key determinant of plant performance in water-limited conditions [12, 13]. Plant water use efficiency decreases strongly when certain atmospheric conditions (high temperatures and low humidity) cause a high evaporative demand.

## Materials and methods

### Turbocharged vs. conventional ICEs

In order to illustrate the advantages of turbocharged and supercharged engines in environments with low substrate (oxygen) concentration, we compared the power output of these engines with conventional ICEs under decreasing oxygen concentration caused by increasing altitude in airplanes. In the case of the supercharged engine, data were obtained on power output with altitude for the Merlin III aircraft during World War II [14]. Power output for conventional ICEs is plotted using an estimate of 3% power loss per thousand foot altitude gain [15].

### C4 vs. C3 photosynthesis

We compared the yield of “supercharged” C4 crops (corn and sorghum) with conventional C3 crops (soybeans and wheat) under varying substrate (CO_2_) concentration. Data for each of the four crops was obtained from a synthesis presented by Long et al. [16] and represents those grown at ambient CO_2_ levels and at elevated CO_2_ levels in chamber experiments. These included 155 measures of soybeans, 211 of wheat, and 14 of corn and sorghum. Solid lines represent a least-squares fit to the data.

### Hybrid electric vehicles vs. ICEs

In order to show how the advantages of hybrid cars increase with variability in driving speed, we analyzed data on gas mileage in model year 2007 vehicles subject to the Environmental Protection Agency (EPA)’s Federal Test Procedure (FTP) cycles. Variance in speed was calculated for both city and highway cycles and mileage information was obtained from model year 2007 vehicles, which have hybrid and conventional counterparts with the same engine: the Toyota Camry, Ford Escape, Nissan Altima, GMC Sierra, and Mercury Mariner. Mileage data was obtained from U.S. Department of Energy and U.S. EPA [17]. Data on speed variance were extracted from city and highway FTP cycles [18].

### CAM vs. C3 crops

To demonstrate how the advantages of CAM photosynthesis depend on variability in transpiration demand, we compared the water use efficiency for C3 and CAM plants with increasing diurnal variability of the vapor pressure deficit. The results were obtained using the Photo3 model [19], which is based on the the Farquhar et al. C3 model [20] and a recently introduced CAM model [21, 22], for one representative species of each photosynthetic type: winter wheat (*Triticum aestivum*) for C3 and prickly pear (*Optuntia ficus-indica*) for CAM. The model was run with a soil moisture of 0.56, soil type of loamy sand, carbon dioxide concentration of 400 ppm; daytime temperature of 303.15 K, solar radiation of 500 W/m^2^, and vapor pressure deficit of 2.89 kPa; and a nighttime temperature of 288.15 K, solar radiation of 0 W/m^2^, and varying nocturnal vapor pressure deficit. Water use efficiency (WUE) is given as a function of decreasing nocturnal vapor pressure deficit, with daytime vapor pressure deficit held constant.

## Results

### Evolution of the substrate concentration mechanism

Over time, both car engines and plant photosynthetic pathways have added components to improve efficiency while leaving the original structures (the ICE and the C3 Calvin cycle) intact. The turbocharger and the C4 carbon pump are added components, which improve performance (engine power output or photosynthetic yield) when low levels of oxygen and carbon dioxide, respectively, limit the efficiency of the core process. The turbocharger adds a turbine and an air compressor to the original ICE. The turbine, driven by the engine’s exhaust gases, powers a compressor which forces more air into the combustion chamber, increasing the available concentration of oxygen (see Fig 2). This improves the volumetric efficiency of the engine and allows a greater power output with less fuel use. Similarly, the C4 photosynthetic pump adds a second carbon fixation process which raises the CO_2_ concentration in the chloroplasts by an order of magnitude. The first pathway functions by carboxylating phosphoenolpyruvate (PEP) to produce a 4-carbon sugar (hence the term C4). The 4-carbon sugar then enters the bundle sheath cell where it is decarboxylated and fixed by RuBisCO in the Calvin cycle (see Fig 2 for a comparison of car and plant components). Because C4 photosynthesis concentrates the CO_2_ at the site of the Calvin cycle, it is able to effectively eliminate photorespiration. This allows the plant to assimilate more carbon with less stomatal opening and water loss.

**Fig 3:**
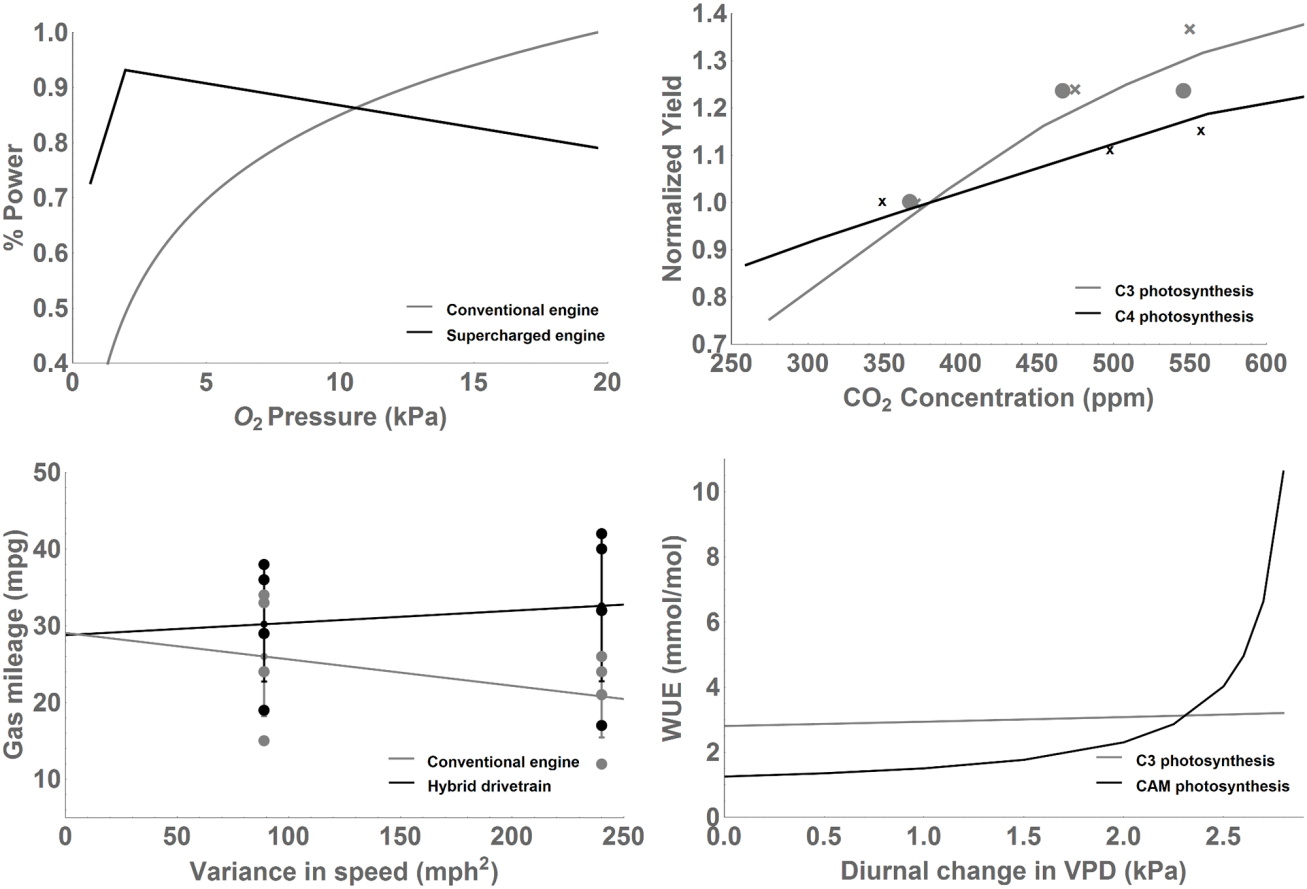
Additional components allow more newly developed photosynthetic systems and car engines to outperform conventional ones under specific conditions. (a) Supercharged engines outperform conventional ICEs with increasing altitude (decreasing O_2_ concentration). (b) Likewise, “supercharged” C4 crops (corn and sorghum combined data) outperform “conventional” C3 crops (soybeans (o) and wheat (x)) with decreasing CO_2_ concentration. (c) Hybrid cars strongly outperform their traditional counterparts under conditions of high variability in driving speed, while they perform similarly under conditions of low variability. (d) In a similar fashion, CAM plants strongly outperform their C3 counterparts in conditions of high variability in vapor pressure deficit, while they are less efficient in the absence of variability.

The turbocharger and the C4 carbon pump developed under limiting oxygen and carbon dioxide conditions, respectively, and both show the greatest advantages over their traditional counterparts in such conditions. Fig 3a compares the power output of the Merlin III, a supercharged jet from WWII, to that of a conventional, non-supercharged airplane, as oxygen pressure changes at altitude. The Merlin III outperforms its conventional counterpart by over 100% at altitudes over two thousand feet, where the oxygen pressure is one-twentieth of that at sea level, but it is limited at lower altitudes due to the power required to run the supercharger. Today, highly developed turbochargers enable decreased fuel consumption and emissions even at sea level and increasingly stringent emissions regulations have caused an increase in the popularity of turbochargers in passenger cars and especially in trucks. Similarly, C4 plants outperform their C3 counterparts most strongly under conditions of low CO_2_. Because of decreased photorespiration they are able to assimilate more carbon and are 2-3 times more water efficient [11]. At the same time, C4 photosynthesis comes with a slight energetic drawback because of the cost of the additional chemical reaction. Thus, the advantages of C4 plants increase strongly at low carbon dioxide levels, and drop off at high CO_2_ levels, where these plants require more solar radiation to assimilate the same amount of carbon (see Fig 3b).

### Evolution of the energy storage mechanism

In a separate strategy, cars and plants both developed energy storage mechanisms, i.e., HEV technology and CAM photosynthesis, which increase efficiency in conditions of high environmental variability. HEVs add a battery and an electric motor to the existing internal combustion engine in order to enable “regenerative braking”—when the brakes are employed, some of the resulting kinetic energy is turned into electricity and stored in the battery. This energy can later be used by the electric motor to assist the internal combustion engine in a dual motor hybrid drivetrain configuration [23–25]. Similarly, the CAM photosynthetic pathway accumulates ‘fuel’ in the form of carbon in the enlarged plant vacuole ‘battery.’ In CAM photosynthesis, stomata open during the night, when transpiration drivers are low, and fix atmospheric CO_2_ as a 4-carbon sugar, typically malic acid, which is stored in the cell vacuole. The malic acid then is decarboxylated during the day and fixed via RuBisCO in the C3 Calvin cycle, which requires light energy (see Fig 2).

Both the HEV and the CAM plant thrive under conditions where efficiency (either fuel efficiency or water use efficiency) is paramount and variability is high. The rise of the hybrid car depended on limiting fuel resources and high demand to improve automobile efficiency. This technology can provide improvements in efficiency up to 34% under stop-start and hilly driving conditions when power demand is variable [26]. At the same time, it introduces the costs of the second power system, the battery, and the more complex control system [24, 27, 28]. Due to this tradeoff, hybrid cars show much better performance than their conventional counterparts under conditions of high variability in driving speed, while they show similar performance under conditions of low driving speed variability (see Fig 3c). Likewise, the CAM pathway is favored in terrestrial environments limited by high costs of daytime stomatal opening due to large water losses, including many arid and semi-arid regions of the world. Because CAM allows the stomata to open at night, when there is a much lower driving force for water loss, CAM water use efficiency is up to six times higher than C3 water use efficiency under typical environmental conditions. Since there is an additional chemical reaction involved, CAM comes with an energetic drawback on the order of 10-20% compared with C3 plants, although this requirement varies depending on what percentage of CO_2_ is taken up at night [29, 30]. Like hybrid cars, which strongly outperform their traditional counterparts when driving speed is highly variable, CAM plants show a major advantage over their C3 counterparts in conditions of high diurnal variability in vapor pressure deficit, a major driver of evaporative demand (see Fig 3d).

## Discussion

Some of the most common modifications to the ICE have striking similarities to the more recently evolved photosynthetic pathways. CAM plants and HEVs differ in a major regard, however, in that the structure of the HEV leaves potential for a redundant power system while that of the CAM plant does not. On the one hand, the CAM plant converts the carbon stored as malic acid back into carbon dioxide before fixing it in the basic C3 Calvin cycle (see Fig 2). On the other hand, parallel hybrid cars use the stored energy in the battery to power the drivetrain electrically through the motor, bypassing the engine entirely. The redundant power system in the HEV, which contains both the ICE and the electric motor, has facilitated the development of the plug-in HEV whose external source of energy comes from both gasoline and electricity [24]. As battery and other electric vehicle technology improves, these systems are becoming a viable option and are replacing the original ICE entirely in some cars, i.e. battery electric vehicles [31]. It would appear that when the means have developed to utilize an entirely new energy source, the stage has been set for the underlying scheme to be usurped by a new one. Indeed, this phenomenon has also been observed in the plant world. As parasitic plants developed the ability to gain carbon from a photosynthetic host, many underwent massive changes in the chloroplast genome leading to loss of photosynthetic function [8]. Parasitic plants, like battery electric vehicles, remain a striking exception to the rule of a highly conserved central component.

The parallels in the evolution of these very different energy production systems provide interesting insight as to how such complex systems are modified over time. In response to moderate environmental pressures, both cars and plants have evolved secondary components to increase the efficiency of the core energy-generating mechanism, while the core mechanism itself remained largely unchanged. Both systems exhibit a high degree of modularity, whereby functional units develop, which are relatively distinct from the surrounding structure [32]. While such modular systems tend to be non-optimal, they are believed to persist because they provide stability and robustness, and show higher adaptability and survival rates under changing environments [32–35]. In both cars and plants, the unchanging central module may lend each system a certain robustness, while the development of auxiliary modules has allowed each system to adapt to changing goals presented by novel environments. The fact that such similar responses can be found in both animate and inanimate systems suggest that a universal mechanism or ‘design principle’ may be at play, e.g. Hartwell et al.; Variano et al.; Bejan et al. [33, 36, 37].

We are likely to observe these dynamics at work in the near future, as, ironically, the pressures of climate change may drive the evolution of plants and cars in very different directions. Climate change is expected to affect plant function through increased levels of carbon dioxide, temperature, and, in many areas, aridity. At a first glance, these changes might be expected to increase the performance of CAM and decrease the performance of C4 relative to C3 photosynthesis [38–41]. However, these outcomes are far from certain and depend on a complex interplay of other factors [42, 43]. In any case, the pressures of anthropogenic climate change are relatively modest compared with historical changes to which the photosynthetic pathway has already been subjected. Considering that photosynthesis has already withstood the test of time, the existing photosynthetic pathways may be expected to adapt to current changes without major evolution. In cars, the story may be different. Modular evolution has historically allowed innovation in automotive technology to adjust quickly to changing goals, yielding turbocharged and hybrid EVs. Yet the prospect of climate change is dramatically increasing pressure to lower carbon dioxide emissions, and perhaps even reduce them to zero. This pressure has lead to the exploration of novel technologies, some of which (including battery and fuel-cell electric vehicles) replace the original ICE altogether [24, 44, 45]. Such technologies have taken more time to develop and may be considered more risky strategies in that they require massive updates to existing infrastructure and manufacturing practices. Compared with plants, which have existed on earth for millions of years, cars are a relatively young technology with interesting possibilities ahead.
